# Numerical Analysis of the Contact Behavior of a Polymer-Based Waterproof Membrane for Tunnel Lining

**DOI:** 10.3390/polym12112704

**Published:** 2020-11-16

**Authors:** Kicheol Lee, Dongwook Kim, Soon-Wook Choi, Soo-Ho Chang, Tae-Ho Kang, Chulho Lee

**Affiliations:** 1 Department of Civil and Environment Engineering, Incheon National University, Incheon 22012, Korea; wlq4619@inu.ac.kr (K.L.); wookdong2@gmail.com (D.K.); 2 Underground Space Safety Research Center, Korea Institute of Civil Engineering and Building Technology, Gyeonggi-do 10223, Korea; soonugi@kict.re.kr (S.-W.C.); thkang@kict.re.kr (T.-H.K.); 3 Construction Industry Promotion Department, Korea Institute of Civil Engineering and Building Technology, Gyeonggi-do 10223, Korea; sooho@kict.re.kr

**Keywords:** waterproof membrane, ground improvement, contact behavior, numerical analysis, interface parameter

## Abstract

Waterproof membranes have higher initial strength, faster construction, and better waterproofing than conventional sheet membranes. In addition, their polymer constituents have much higher interfacial adhesion and tensile strength than those of conventional materials. However, despite their advantages, waterproof membranes are not widely used in civil construction. This study evaluates the material properties and interface parameters of a waterproof membrane by considering the results of laboratory experiments and numerical analysis. Since the contact behavior of a membrane at its interface with shotcrete is important for understanding the mechanism of the support it offers known as a shotcrete tunnel lining, modeling should adopt appropriate contact conditions. The numerical analysis identifies the suitability and contact conditions of the waterproof membrane in various conditions.

## 1. Introduction

Waterproofing both new and existing underground structures is an economical and effective way to increase their design life. It can also improve the safety and structural integrity of these structures and minimize the maintenance costs resulting from environmental changes. Waterproofing can generally help a structure function during its design life. In addition, given that the groundwater entering underground works or tunnel excavation sites may degrade the performance and durability of structures by causing ground subsidence or settlement, structural waterproofing (provided by a waterproof layer) is an important consideration [1]. Therefore, a sheet membrane, such as PVC (polyvinyl chloride), is generally used alongside grouting to waterproof excavated tunnel sections. A shotcrete lining is applied on top of an installed waterproof sheet membrane to protect it. However, any damage to the lining caused during construction can lead to leakage.

Guidance from The International Tunneling Association [2] regarding waterproof membranes states they should generally be 3–5 mm thick, which is much thinner than a shotcrete layer. Waterproof membranes have been reported to be able to prevent the penetration of water or moisture [2]. To clearly understand the performance of a waterproof membrane, both field and laboratory tests are required [3]. The cohesion, initial strength, and workability of membranes make them suitable waterproof materials [2]. The combined shotcrete–membrane system can then function as a complex composite structure [4]. However, few recent reports have considered the properties of the contact surface or attachment surface of waterproof membranes [4,5,6,7,8,9]. The European Federation of National Associations Representing for Concrete (EFNARC) proposed thin spray-on liners (TSLs)—polymer-based, permanent supporting materials—for underground structures and tunnels [10]. A TSL is also generally 3–5 mm thick (but can reach a maximum thickness of 10 mm). In addition, a TSL has a very similar composition for a waterproof membrane but generally shows much higher structural performance because of its function for supporting material. Therefore, criteria for the TSL by EFNARC (European Federation of National Associations Representing for Concrete) [10] is focused on its mechanical properties while criteria for the waterproof membrane by ITA (International Tunneling and Underground Space Association) [2] is focused on its waterproofing capability and chemical resistance.

In previous research by Park et al. [11], mechanical and interfacial properties of the waterproof membrane, which is used in this study, were well reported. In previous research, waterproofing the membrane prototype based on EVA (ethylene-vinyl acetate) polymers were found to be satisfactory for use as waterproofing and rock support, as they satisfied minimum performance requirements suggested by ITA [2] and EFNARC [10]. In addition, Lee et al. [8] used a simple numerical method to compare the performance of the waterproof membrane with conventional TSL as a support material and reported that contact condition between membrane and concrete lining had a relatively large effect on tensile behavior.

In this study, the structural performance of the waterproof membrane was to be reviewed by the analysis techniques used in the tunnel design. For this, the construction location of the waterproof membrane was set as a case, and the tunnel cross-section analysis was performed to analyze the stability. Analysis Case 1 had no waterproof membrane. Case 2 had the membrane attached to the intrados surface of the shotcrete lining, assuming that it provided only waterproofing and no structural support. In addition, Case 3 had the membrane in the middle of the shotcrete lining. Cases 2 and 3 had two types of a contact condition set (tangential and cohesive behavior), and the appropriate condition was analyzed. The stability of a section of tunnel structure with a membrane and shotcrete lining was also assessed based on the permissible values of flexural compressive stress, flexural tensile stress, and shear stress.

## 2. Characteristics of the Waterproof Membrane

### 2.1. Composition of the Waterproof Membrane and Water Tightness

The composition of the waterproof membrane used in this study is shown in Table 1. Prototype 1 is a liquid-type waterproof membrane composed mainly of alumina cement and calcium sulfo-aluminate, and a 3:1 weight mixing ratio of liquid EVA polymer and powder material. Prototype 2 is a waterproof membrane that is mixed with a powdered EVA polymer and other powdered materials, and is designed to mix with water in a ratio of 1:3 during construction. Prototype 2 is relatively cheaper and quicker to prepare than Prototype 1, and its general mechanical properties is better than Prototype 1. However, as a result of X-ray and CT scanning conducted by Park et al. [11], both membrane prototypes were kept waterproof at a water pressure of 5 bar for 28 days. Other performance criteria were also satisfied with the performance range of TSL by EFNARC [10]. In this study, a waterproof membrane was prepared according to the proportion of Prototype 2, and tests and numerical analysis were performed.

### 2.2. Mechanical Properties of the Waterproof Membrane

**Figure 1:**
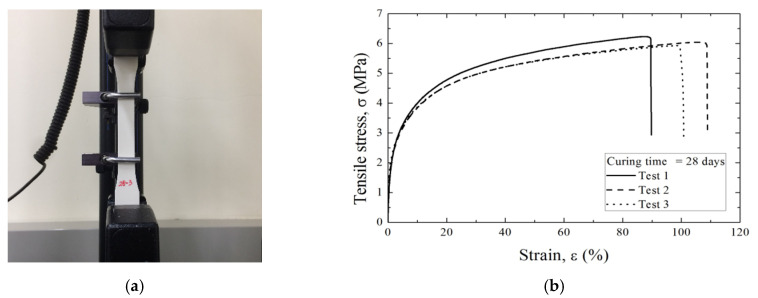
Tensile testing: (**a**) setup and (**b**) results of three tests for samples cured for 28 days.

The material properties of the waterproof membrane were assessed in tensile tests conducted according to the ASTM-D638 standard [12]. Membrane specimens with a thickness of 3 mm were made, following the Type-4 specification of the ASTM standard. Figure 1a shows the tensile test procedure and results. Specimens formed in custom-made molds were tested after 28 days. The tensile stress–strain curves in Figure 1b show plastic behavior from a strain of about 2% and a mean tensile stress of 6 MPa.

The test results established the mechanical properties of the membrane for use in the numerical analysis. The backtracking method ensured that the analytical model matched the actual test specimen. It conducted the analysis as specified by the ASTM D-638 standard using a range of mechanical properties of the modeled material. When the modeled mechanical properties gave results sufficiently close to the actual test results, the analysis was terminated, and those properties were assigned to the material. The studied waterproof membrane showed perfectly plastic behavior, agreeing with Holter’s [4] observation of perfect plasticity in a polymer-based waterproof membrane. Therefore, the mechanical properties of the membranes used in numerical analysis were elastic modulus, plastic parameter values, and Poisson’s ratio.

**Figure 2:**
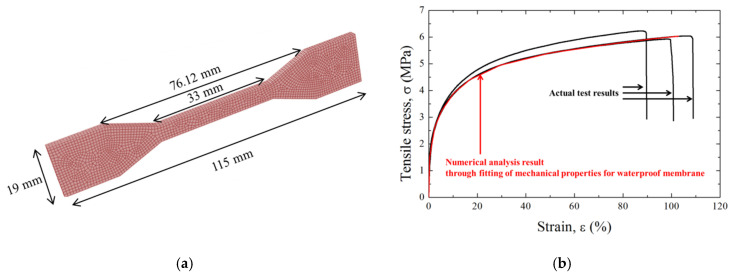
Numerical analysis: (**a**) tensile test model and (**b**) results.

The analytical model illustrated in Figure 2a produced the results in Figure 2b by fitting the mean of the three empirical tensile tests using the ABAQUS numerical program [13]. The elastic modulus significantly influenced the initial slope of the tensile stress–strain curve. The Poisson’s ratio did not significantly affect the analytical results, and the value for a waterproof membrane used by Lee et al. [8] was used here. The plastic parameter values affected the interval of yielding after the elastic section. Table 2 summarizes the mechanical properties of the membrane with respect to the analytical results in Figure 2b. The density of the membrane was directly measured from the weight of the empirical specimen because changing the density in the analysis did not affect the tensile stress–strain curve.

## 3. Contact Properties between the Membrane and Shotcrete

The contact properties of the membrane on shotcrete are an essential part of structural stability analysis. They were numerically analyzed here by comparing two models of contact (tangential and cohesive behavior) between the membrane and shotcrete. The tangential and cohesive behaviors were implemented numerically through direct shear and linear behavior tests.

### 3.1. Tangential Behavior Model

The Coulomb contact model is commonly used for the interface of two contact surfaces. It is based on the coefficient of friction, and assumes ideal behavior for an object with a contact surface. Therefore, slip occurs in proportion to the magnitude of the normal stress acting on the object [14].

The ABAQUS analysis program defined the model as having tangential behavior, and the friction surface characteristics were defined by selecting a specific coefficient of friction. Since the two surfaces come into contact with each other, shear forces act on the contact interface with the following characteristics [13]: the critical friction stress varies with the pressure of the contact surface, the critical shear stress (τ_cr_) is related to the friction coefficient (μ) and contact pressure (P) (τ_cr_ = μP), the friction coefficient is a function of conditions such as relative slip velocity, pressure, and temperature, and the basic setting of the friction model is to approximate ideal behavior and allow a small elastic slip before an irreversible slip occurs.

**Figure 3:**
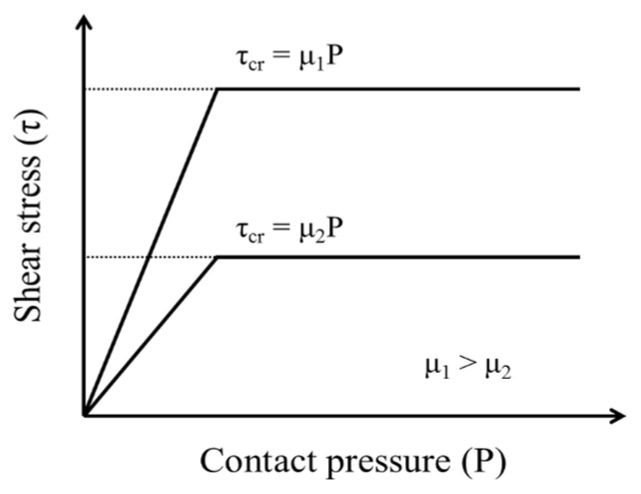
Coulomb friction model (modified after Kim [14]).

Using this Coulomb model, Figure 3 plots the increasing shear stress with respect to increasing contact pressure up to the critical shear stress, above which the shear stress does not increase and remains constant regardless of the contact pressure. The critical shear stress level can, however, be raised by increasing the friction coefficient.

#### 3.1.1. Direct Shear Test

**Figure 4:**
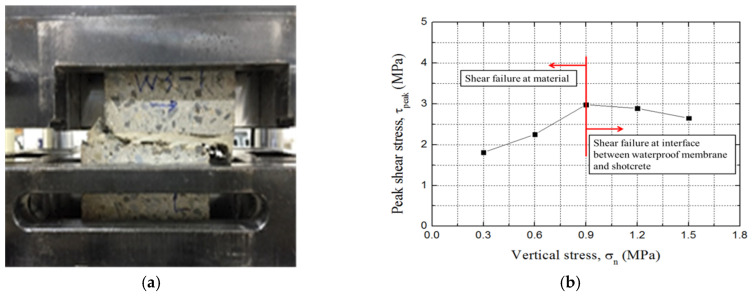
Direct shear test: (**a**) setup and (**b**) results.

The tangential behavior was analyzed here based on the direct shear test results for this waterproof membrane and shotcrete obtained by Park et al., using the setup in Figure 4a [11]. A 7.5-cm specimen of shotcrete was cured in a frame for 28 days, and the membrane was then applied to a thickness of 3 mm. When the membrane had hardened, the sample was cured for a further 28 days before the shear test was performed.

Figure 4b plots the direct shear results with respect to vertical stress. It shows that the peak shear stress did not increase after the vertical stress had increased above a certain value (0.9 MPa). This was because the shear failure of the water membrane itself occurred at low vertical stress, and the shear failure of the interface between the membrane and the shotcrete occurs mainly at high vertical stress [9].

#### 3.1.2. Numerical Analysis Based on the Direct Shear Test

**Figure 5:**
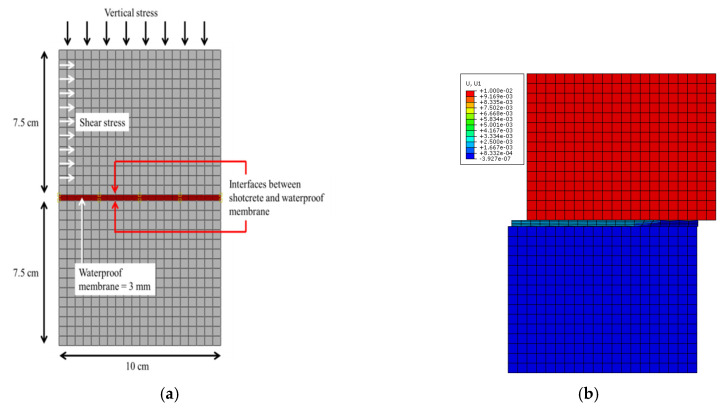
Direct shear test: (**a**) model and (**b**) visualization of uniform horizontal displacement.

Figure 5 shows the analytical numerical model and a visualization of the direct shear analysis. The modeled specimen was 15.3 cm thick, consisting of a 3-mm membrane between the two shotcrete layers, which are each 7.5 cm in thickness. The analysis comprised two-dimensional, “plane-strain” modeling elements, and the boundary conditions of the lower shotcrete layer had its bottom and side fixed. The upper shotcrete layer was displaced from left to right as in the actual direct shear test so that the shear force was generated at the friction surface.

#### 3.1.3. Estimation of Parameters for Tangential Contact Behavior

**Figure 6:**
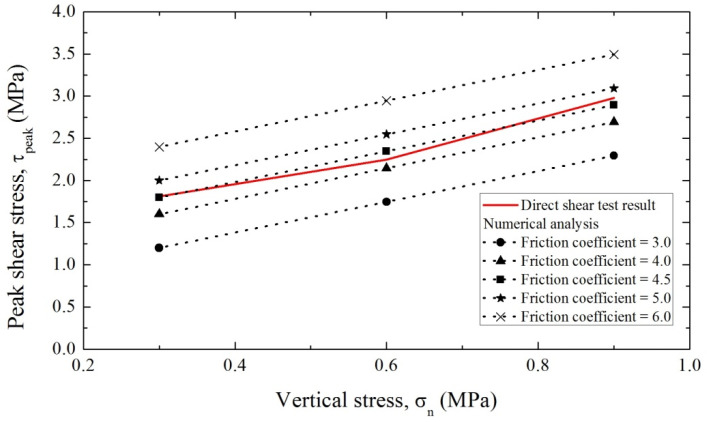
Results of numerical analyses with different friction coefficients for fitting the tangential behavior of the waterproof membrane.

Figure 6 shows the numerical results for measuring the tangential behavior of the membrane. Results are shown for various values of the friction coefficient (3.0–6.0), which is a parameter of tangential behavior. The measured direct peak shear stress values increased linearly with vertical stress, following the numerical result for a friction coefficient of 4.5 most closely.

### 3.2. Cohesive Behavior Model

Numerical analysis includes cohesive behavior as part of the surface interaction dynamics [13]. In a cohesive model, “cohesive behavior” is regarded as the linear elastic region encountered during a period of tensile force applied at an interface, and this behavior occurs before the onset of surface separation. This elastic behavior can be expressed using elastic constitutive equations for normal and shear stresses when normal and shear separation occur at the contact surface. “Damage behavior” refers to the simulation of damage and failure of the bond between two cohesive surfaces. Damage and failure occur when the contact pressure reaches a specified failure criterion. The damage and failure mechanisms have two parts: Initiation threshold and evolution trajectory, which should both be specified in a cohesive model. Without a specific path of damage evolution, even if the damage initiation threshold is input, there is no defined way it would affect the cohesive model. In addition, a cohesive surface has one damage initiation criterion and one damage evolution law, meaning that multiple damage mechanisms cannot be inputted. The “evolution energy,” also termed “fatigue energy,” is related to the linear or exponential ductility behavior of the interface after its damage and failure.

The behavior of a cohesive contact surface typically progresses via traction to separation. The initially cohesive behavior represents the maintaining of contact while a separating force is applied to the contact surface. For surfaces with high cohesive stiffness, a large shear stress must be generated to separate the interface. Damage initiation is the state in which the surfaces begin to lose contact. After damage occurs, the behavior is determined by the evolution energy, and complete separation occurs when the contact energy of the contact surface disappears.

#### 3.2.1. Linear Block Support Tests

**Figure 7:**
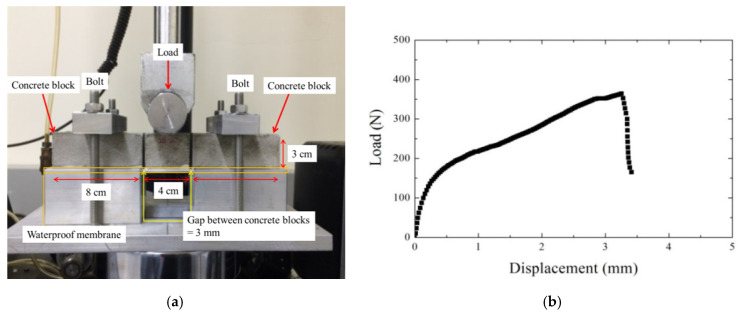
Linear block support test proposed by EFNARC [10]: (**a**) setup and (**b**) results.

The waterproof membrane had a strong cohesive force, and, thus, had significant bonding strength on the shotcrete lining. To evaluate the contact condition between it and the shotcrete, the linear block support test proposed by EFNARC [10] was performed, using blocks and shotcrete with the dimensions shown in Figure 7a. The Linear Block Support (LBS) test is carried out with three concrete blocks. Two side blocks are 8 cm long and the middle block is 4 cm long. Each block has a cross-sectional shape of a 4 cm × 3 cm and the spacing between blocks is 3 mm. The waterproof membrane is applied to the bottom of concrete blocks and then cured for 28 days. A load was applied to the middle block to induce adhesion failure between the blocks and the membrane layer. The membrane layer was fixed to the side blocks with bolts. The tests were carried out three times, and the highest measured cohesive strength is plotted in Figure 7b, which shows failure occurring at about 400 N with a mean displacement of 3.22 mm.

#### 3.2.2. Numerical Analysis Based on the Linear Block Support Test

**Figure 8:**
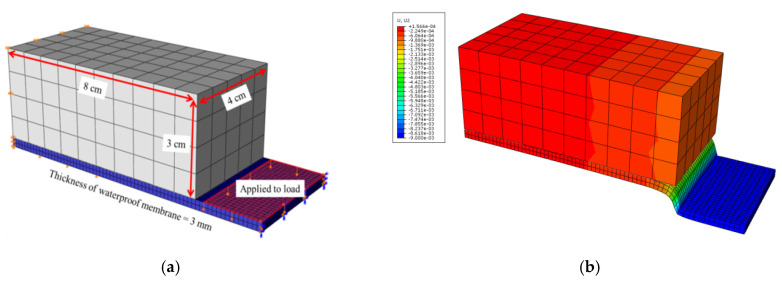
Linear block support test: (**a**) model and (**b**) visualization of uniform vertical displacement.

The waterproof membrane was modeled in three dimensions using a rock block with dimensions of 8 cm × 4 cm × 3 cm. The model of the linear block test used the conditions of symmetry around the middle rock block to improve analytical efficiency. The distance between the rock blocks was set as 3 mm, as in the empirical test, and a fixed boundary condition was applied to the end of the block. The middle rock block responsible for the loading was not modeled in the analysis, and was, instead, considered as a direct load on the surface of the waterproof membrane. Figure 8a shows that, at the start of the analysis, the waterproof membrane under the load had a constant displacement.

Figure 8b shows the waterproof membrane detached from the rock block. Before the contact surface was damaged, the contact state was maintained owing to the cohesive properties defined on the contact surface. The contact state diminished gradually as the damage increased.

#### 3.2.3. Estimation of Parameters for Cohesive Contact Behavior

**Figure 9:**
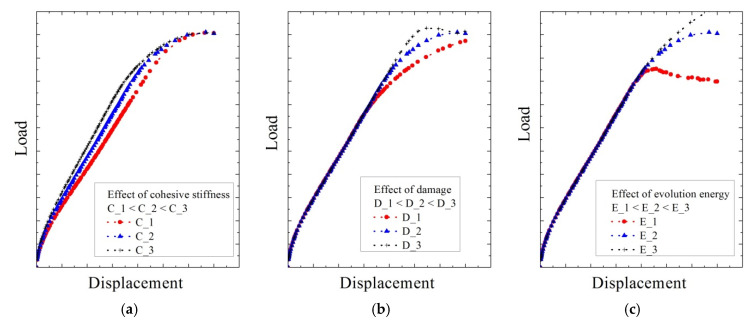
Numerical analysis results for linear block support tests obtained by varying the parameters of (**a**) cohesive stiffness, C, (**b**) maximum stress of damage initiation, D, and (**c**) evolution energy, E.

Figure 9 shows the behaviors of parameters for cohesive behavior (cohesive stiffness, maximum stress of damage initiation, and evolution energy) in linear block support tests reported by Lee et al. [8]. The red line indicates when a relatively lower value is used compared to the target value, and the gray line indicates when a higher value is used. In addition, a red and gray line shows the tendency, according to each cohesive contact value instead of the load-displacement relationship for a specific value. As shown in Figure 9, increasing the cohesive stiffness increased the slope of the load–displacement relationship in the analytical model, but the load required for damage was unaffected (Figure 9a). Figure 9b shows the change in the maximum load at which the damage to the contact surface began. The initial linear slope increased to the same value in each case, but damage occurred at a lower load when the maximum stress of damage initiation was small. Figure 9c shows the evolution energy required for removal of the contact surface. The initial slope and the damage point were similar, but, for a lower evolution energy, the load was not increased even if the displacement increased.

In order to obtain a cohesive contact property of the membrane, the analysis was performed by trial and error. The slope of the initial curve was determined by “cohesive stiffness,” the maximum load and the rear slope were determined by “Maximum nominal stress at damage initiation,” and the point of load reduction was determined by “evolution energy.” Although the three variables were not completely independent, this was solved through a large number of simulations. The process was performed until it reached within ±5% of the range similar to the result of Figure 7b. Table 3 summarizes the optimal fit for cohesive stiffness, maximum stress of damage initiation, and evolution energy, showing the contact characteristics of the waterproof membrane derived from the comparison of empirical and numerical results.

### 3.3. Analysis of General Beam Members in Different Contact Conditions

**Figure 10:**
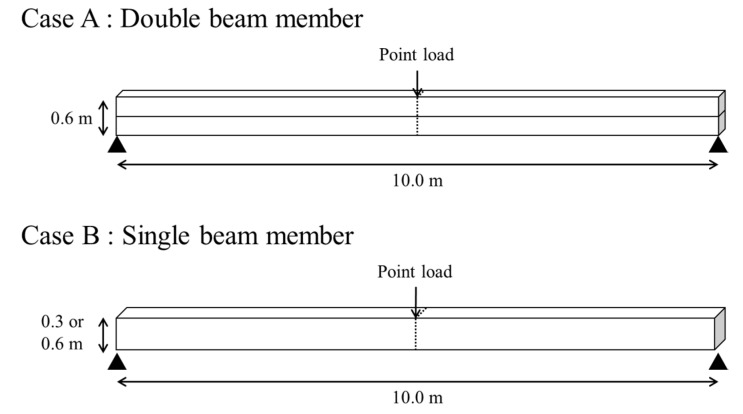
Analysis of the contact behavior of general beam members.

Numerical analysis for a general beam member was performed simply to check differences in the contact conditions. Figure 10 shows the analysis and modeling conditions. First, two beam members (each 0.3-m thick and 10-m long) were modeled. The contact conditions were “Tie” for complete contact, “Tangential behavior” for general shear conditions, and “Cohesive behavior” for adhesion of contact surfaces. No waterproof membrane was inserted inside because this analysis sought to confirm simple contact behavior. A point load was applied in the center of the upper beam member, and the displacement and force were analyzed accordingly. As a boundary condition, both ends of the lower beam member were fixed. For comparison, single beam members (with thicknesses of 0.3 and 0.6 m) without contact conditions were similarly analyzed. The material properties of the beam member were elastic modulus of 25.8 GPa and Poisson’s ratio of 0.2, and were based on those of plain concrete. The concrete’s unit weight was ignored because gravity was not considered.

**Figure 11:**
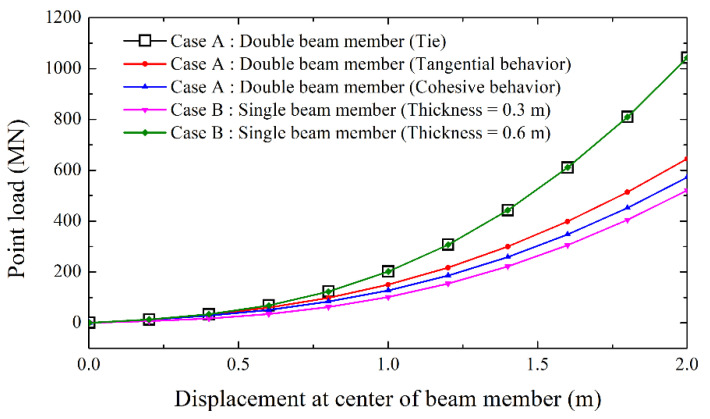
Analysis of contact behavior of general beam members.

The results of the analysis are shown in Figure 11. The need for more force to reach the same displacement means that stiffness of the beam member is greater than others. Especially in double beam cases, higher stiffness means that the behavior of the contact surface relatively appears in the form of a composite structure.

The deformation occurred small in the order of tie, tangential, and cohesive, which means that the tie withstands greater strength. The behavior of complete contact simulated in the “Tie” condition very closely followed the results for a single beam member of equivalent thickness, while tangential and cohesive behavior showed relatively low poor performance with greater deflection at a given load. These results indirectly confirmed the interface characteristics, specifically the compressive force, for the different contact conditions, and the influence on the structure receiving the tensile force, such as the tunnel lining that must be additionally confirmed. This is mentioned later in Section 4.

## 4. Implementation of a Membrane and Shotcrete Lining in a Tunnel

### 4.1. Numerical Analysis Procedure

**Figure 12:**
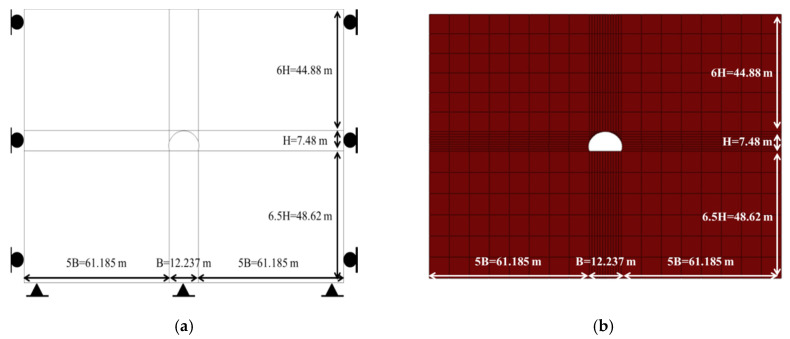
Modeling of analytical ground. (**a**) Schematic diagram and (**b**) mesh formation.

Figure 12 shows the ground in which the tunnel lining is constructed, and follows the analytical model given by Park et al. [15]. The ground is weathered rock, and the tunnel is located 44.88 m below its surface. The ground model either side of the tunnel extends five times of the tunnel width (B) (i.e., 5B = 61.185 m). The height of the ground above the tunnel (44.88 m) is six times the tunnel height (H), and, below the tunnel, the ground extends 6.5H (i.e., 48.62 m).

The side and bottom ground boundaries are fixed. The ground to the side of the tunnel allows vertical movement but not horizontal movement, and the ground below the tunnel is fully fixed and assumed to be a support layer that only loads due to gravity, which is applied to the whole model.

Stability analysis considers a low-level tunnel surrounded by the rock of grade V in the Rock Mass Rating scheme. This grade is unfavorable for tunneling, and requires much reinforcement prior to installing the shotcrete lining. The shotcrete lining is designed as an arch comprising curved and linear segments. It is preferable that the various curved and linear portions have a common tangent at their meeting points in order to form a smooth arch [15].

**Figure 13:**
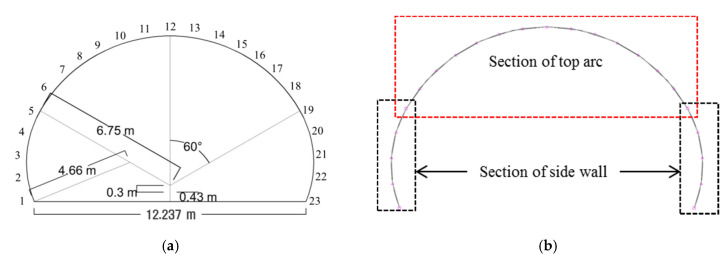
Modeling of analytical tunnel lining. (**a**) Schematic diagram and (**b**) mesh.

Therefore, the tunnel lining in this study is composed of two smaller circular arcs (radius 4.66 m) as side walls and a larger circular arc (radius 6.75 m) as the tunnel roof (Figure 13). This gives a simple, yet realistic, two-dimensional analysis model to study the contact area of the waterproof membrane. The numbering in Figure 13 represents the locations of the nodes with nodes 5–19 representing the top arc, and nodes 1–5 and 19–23 representing the two side walls. The shotcrete lining was set as “Deformable-wire” in ABAQUS to calculate the axial shear forces and moments of beam members [13].

The numerical analysis considers the material properties listed in Table 4. Data for the weathered rock ground are from the literature [15]. The analysis uses an elasto-plastic model with a nonlinear plastic region because weathered rock has similar Mohr–Coulomb characteristics, including cohesion and a friction angle. The material properties of the shotcrete lining are assumed to be those of plain concrete (without reinforcing bars), whose unit weight is 23.5 kN/m^3^ and material strength (*f_ck_*) is set to 24 MPa. The cross-sectional for the plain concrete is designed by the allowable stress design method, and it is common to calculate the stress due to the working load using linear elastic theory. Therefore, the properties of the shotcrete lining are listed in Table 4, and its elastic modulus (*E_c_*) is calculated using Equation (1), according to the concrete structural standard [16].

(1) $$E_{c}(MPa)=8500\sqrt[{3}]{f_{cu}(MPa)}$$

where *f_cu_* is the average compressive strength of the concrete, *f_cu_* = *f_ck_* + Δ*f*, and Δ*f* is defined as 4 MPa when *f_ck_* is less 40 MPa and 6 MPa when *f_ck_* is 60 MPa or more.

**Figure 14:**
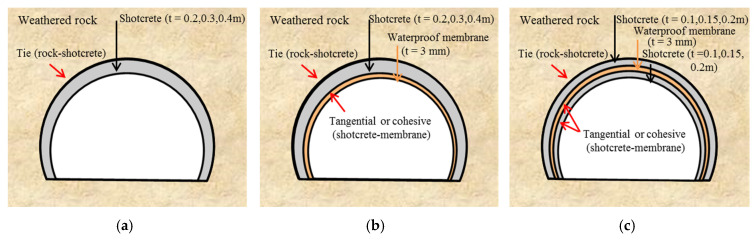
Setups for the stability analysis of tunnel linings. (**a**) Case 1: Shotcrete lining on a rock. (**b**) Case 2: Waterproof membrane attached to the bottom of shotcrete, and (**c**) Case 3: Membrane inside the shotcrete.

The following test cases confirm the effect of a waterproof membrane on the shotcrete lining (Figure 14). Case 1 has only the shotcrete lining installed in the ground with no waterproof membrane. It is tested at thicknesses of 0.2, 0.3, and 0.4 m. In actual construction, the shotcrete is placed in close contact with the ground, so the contact surface of the ground and the shotcrete is set as ‘constraint-tie,’ which is the interfacial characteristic corresponding to the complete contact (Figure 14a). Case 2 includes a 3-mm thick waterproof membrane attached to the intrados surface of the shotcrete lining (Figure 14b). The numerical analysis considers the contact surface of the membrane and shotcrete as showing tangential or cohesive behavior characteristics. Case 3 has the membrane inserted inside the shotcrete lining, splitting it into two equal thicknesses (Figure 14c). The contact surface characteristics are shown in Case 2.

### 4.2. Results of Numerical Analysis

**Figure 15:**
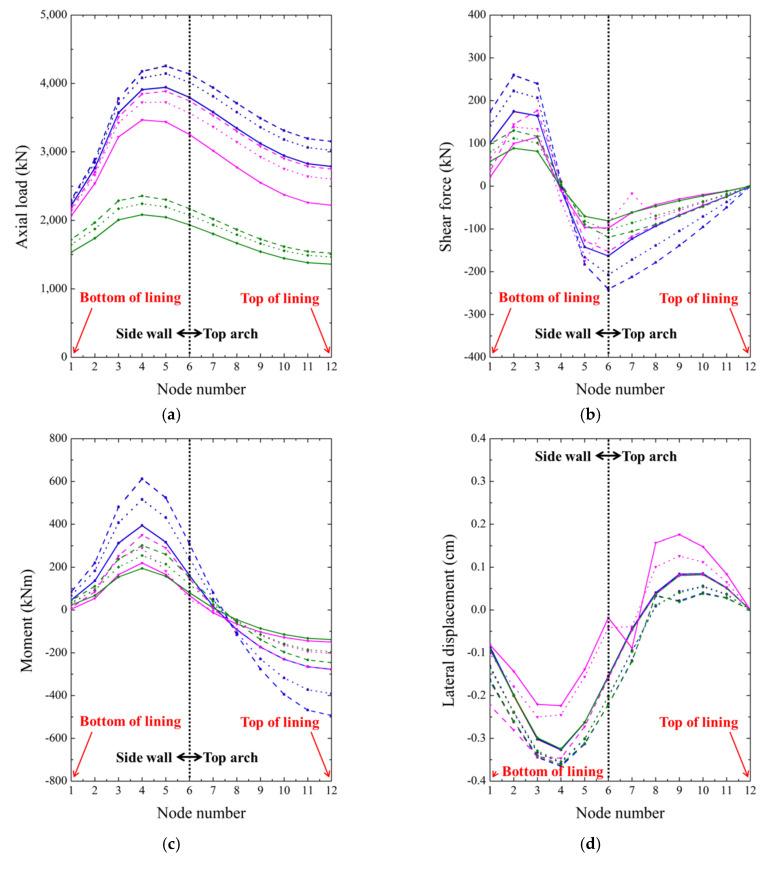

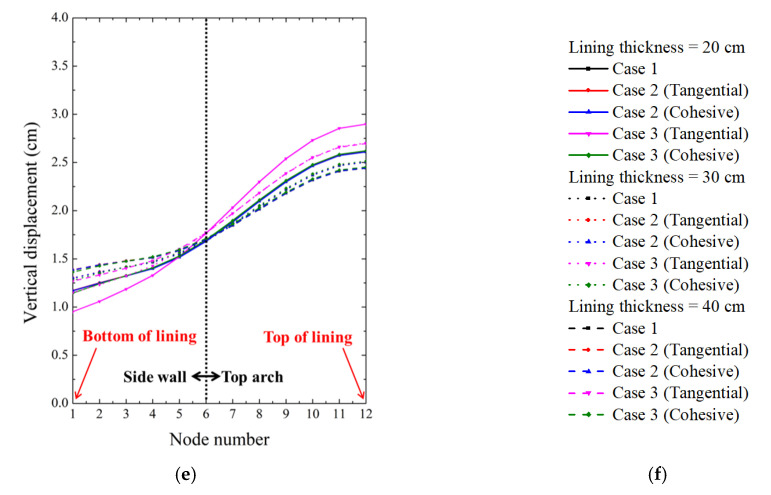
Results for various tunnel-lining cases: (**a**) axial load, (**b**) shear force, (**c**) moment, and (**d**) lateral, (**e**) vertical displacement and (**f**) legend.

The node positions of the shotcrete beam members are shown in Figure 13. The axial load, shear load, moment, and lateral and vertical displacement measured at each node are shown in Figure 15. The maximum values in each case are listed in Table 5. The axial load is defined as the force applied on a shotcrete member directly along an axis of the member. The shear force is the force acting in the vertical direction of the member and the moment means the bending stress that occurs in the member when short bending occurs. In all the analysis results, symmetry is based around the uppermost point of the top arc (node 12). Therefore, only nodes 1 to 12 are shown on the graph, and node 1 represents the bottom end of the lining, and node 12 represents the top end of the lining. Furthermore, as mentioned above, nodes 1~6 are at side walls, and nodes 6~12 are at top arcs.

Cases 1 and 2 under two contact conditions show very similar results for each of the parameters in Figure 15. Therefore, the black line for Case 1, and the red and blues lines for Case 2 in tangential and cohesive contact behavior, respectively, overlap each other, and only the blue line is visible. This is also confirmed from the maximum values in Table 5. These results show that, when the waterproof membrane is attached to the bottom of the shotcrete lining, it has no significant effect on the analysis results, and the contact conditions are not required in the modeling. The subsequent analysis, therefore, excludes Case 2, and Case 1 (with no membrane) is compared only with Case 3 (with the membrane within the shotcrete lining), according to the contact conditions (tangential and cohesive).

Figure 15a–c shows that the axial load increases from the bottom of the shotcrete lining up the side wall, and then gradually decreases in the top arc. The shear force was symmetrical with respect to the upper arch, and the largest value was measured at the side wall. It is zero at the uppermost point of the top arc (node 12). Going up the side wall, moment increases positively from near zero. When ascending the top arc, it begins to decrease, and changes direction (i.e., becomes negative) from node number 7.

In all cases, increasing the thickness of shotcrete lining significantly alters the axial load, shear force, and moment. Under cohesive contact between the shotcrete and membrane, the axial load, shear force, and moment are smaller than under tangential behavior, and they are smaller than in Case 1 at the same thickness. Measurement for Case 1 considers individual shotcrete lining members of 0.2, 0.3, and 0.4 m, while the results for Case 3 are measured in an upper shotcrete lining layer of thickness 0.1, 0.15, or 0.2 m. Therefore, a difference may occur owing to the member thickness, but if the contact condition is tangential, the generated force of tunnel lining is measured excessively than when in cohesive behavior. It is, therefore, necessary to consider tangential contact behavior as, in comparison to cohesive contact behavior, it shows more similar results to the single member of Case 1.

Figure 15d,e depicts the lateral and vertical displacement. Case 3 with tangential behavior shows an unusual result. In the lateral behavior, various changes occur depending on the thickness. Therefore, it is not appropriate to set the contact condition as tangential when inserting the membrane inside the shotcrete.

### 4.3. Examination of Shotcrete Lining According to the Waterproof Membrane Construction

To analyze the performance of the shotcrete lining for different waterproof membrane conditions, the following parameters used in tunnel lining stability analysis are calculated for all cases: Flexural compressive stress (*f_c_*), flexural tensile stress (*f_t_*), and shear stress (*V_c_*). Equation (2) gives the first two (*f_c_* is calculated for a positive moment over section modulus ratio, *M*/*Z*, and *f_t_* is calculated with a negative *M*/*Z* value), and Equation (3) gives the last. The calculations yield the forces of each element of the shotcrete beam members.

(2) $$f_{c}\;or\;f_{t}=P/A\pm M/Z$$

(3) $$V_{c}=V/A$$

where *P* is the axial load, *A* is the section area, and *V* is the shear force.

These three stress parameters are set to allowable values that depend on the properties and thickness of the shotcrete lining members. A high value for any of them can jeopardize tunnel stability, so stability analysis focuses on reducing them.

**Figure 16:**
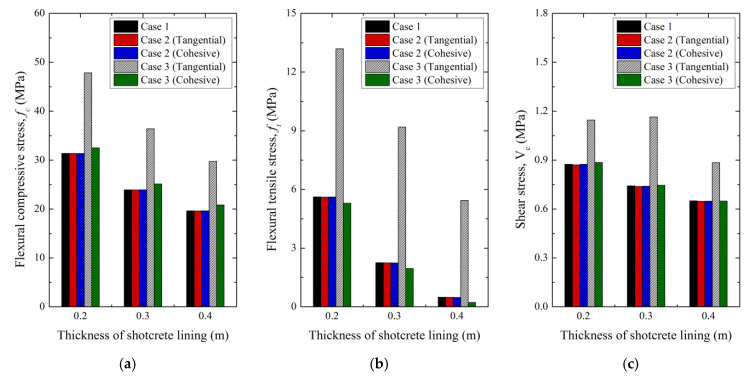
Result for each case for (**a**) flexural compressive stress, (**b**) flexural tensile stress, and (**c**) shear stress.

Figure 16 shows values for each stress and, as in the previous analysis, there is no significant difference between Case 1 (without a membrane) and Case 2 (with a membrane at the bottom of the shotcrete). In Case 3, with tangential contact behavior, there are significant differences in all stress analysis results. As a result of calculating the intensification factor according to Equation (4), the flexural compressive stress increases from 51.34% to 52.50%, flexural tensile stress increases from 134.72% to 1015.33%, and shear stress increases from 31.00% to 35.99% when compared with the results for Case 1. In Case 3 with cohesive contact behavior, the flexural compressive stress increases by 3.57%–6.09%, and the flexural tensile stress decreases by 5.80%–54.60% relative to Case 1. The equivalent changes of shear stress are a 1.30% increase for a 0.2 m-thick lining, an increase of 0.39% at 0.3 m, and a decrease of 0.14% at 0.4 m.

(4) $$E=\frac{\sigma_{Case3,t,contact}-\sigma_{Case1,t}}{\sigma_{Case1,t}}\times 100(\%)$$

where *E* is the intensification factor, *σ* is the relevant stress (flexural compressive, flexural tensile, or shear), contact is either tangential or cohesive, and *t* is the thickness of the shotcrete lining.

These results show that attaching the waterproof to the bottom of the shotcrete does not affect the tunnel stability analysis. When it is inserted inside the shotcrete lining, it is not appropriate to apply tangential contact behavior because it results in unrealistic values. Considering properties such as the cohesion or adhesion of the waterproof membrane, it is appropriate to set the contact condition as cohesive. In addition, for cohesive behavior, flexural compressive stress increases and flexural tensile stress decreases. Therefore, it is necessary to determine the appropriate shotcrete lining thickness and any issues regarding internal insertion during tunnel design while also paying proper attention to the ground conditions and tunnel shape.

## 5. Conclusions

This study analyzed the properties and contact behavior of a waterproof membrane used for waterproofing existing shotcrete tunnel linings. Experimental and numerical tests examined whether the membrane itself could contribute to the tunnel’s stability. The results are as follows.

(1) Tensile tests established that the membrane shows elastic and plastic behavior, so an elasto-plastic model was applied in the numerical analysis. Direct shear tests and linear block tests determined the contact condition between the membrane and shotcrete. The former obtained a tangential behavior model, and the latter obtained a cohesive behavior model.
(2) Analysis of the forces acting on the shotcrete lining (axial load, shear load, and moment) and the lateral and vertical displacements revealed insignificant differences regardless of the application of a waterproof membrane and contact condition. The stresses used in tunnel stability analysis (flexural compressive, flexural tensile, and shear) were similarly unchanged. Therefore, attaching the membrane to the bottom of the shotcrete does not affect the overall tunnel stability, so it need not be considered at the design stage.
(3) Inserting the membrane inside the shotcrete greatly influenced the forces acting on the shotcrete, its displacement, and the stresses used to assess tunnel stability. The different contact conditions gave significantly different results. Those for tangential behavior were discounted as abnormal, and excessive stress was generated in comparison to analysis results in which the membrane was not applied.
(4) The non-conforming results for tangential behavior and the properties of the waterproof membrane itself (such as cohesion and adhesion) indicate that the contact between the shotcrete and membrane is cohesive. With regard to the overall tunnel stability, the membrane increases flexural compressive stress and decreases flexural tensile stress. Shear stress increases or decreases, depending on the shotcrete’s thickness. Therefore, tunnel design must consider the shotcrete structure when planning the placement of a waterproof membrane.
(5) Tunnel design must rely on numerical analysis because actual experiments are impossible in reality. However, results can be verified using model experiments. Further to the work of this study, which considered only a waterproof membrane of 3 mm thickness, subsequent studies are expected to test different membrane thicknesses.

## Figures and Tables

**Table 1:** Chemical composition of the sprayed waterproof membrane (weight %) [11].

Materials	Prototype 1 (Two-Component)	Prototype 2 (One-Component)
Alumina cement	30.0	15.0
Calcium sulfo-aluminate	30.0	-
Calcium carbonate	19.5	14.1
Slag	15.0	-
Nano silica	4.0	-
Lithium carbonate	0.1	-
Citric acid	0.3	-
Anhydrous gtpsum	-	5.0
Hydroxypropyl methylcellulose	0.5	-
Antifoaming agent	0.3	-
Aluminum hydroxide	-	10
Thickener	-	0.85
Promoter	-	0.05
Synthetic fiber	0.3	-
Powder-type EVA (ethylene-vinyl acetate) polymer	-	55.0

**Table 2:** Mechanical properties of the waterproof membrane.

Material Property	Density (kg/m^3^)	Elastic Modulus (MPa)	Poisson’s Ratio
Value	1070	486.54	0.3

**Table 3:** Cohesive contact properties of the studied waterproof membrane.

Cohesive Contact Property	Value
Cohesive stiffness	2.7 GPa
Maximum nominal stress at damage initiation	1.2 MPa
Evolution energy	1.0 kJ/m^2^

**Table 4:** Material properties of the ground (Park et al. [15]) and plain shotcrete.

Material	Density (kN/m^3^)	Elastic Modulus (MPa)	Poisson’s Ratio	Cohesion (kPa)	Friction Angle (°)
Weathered rock	22.0	250	0.3	50	35
Shotcrete lining	23.5	25,800	0.2	-	-

**Table 5:** Maximum values of various parameters found in numerical analyses for each case.

Case	Thickness of Shotcrete Lining (m)	Contact Property	Max. Axial Load (MN)	Max. Shear Force (MN)	Max. Moment (MNm)	Max. Displacement (cm)	
						Lateral	Vertical
1	0.2	-	3.945	0.175	0.394	0.327	2.609
	0.3	-	4.146	0.222	0.516	0.357	2.501
	0.4	-	4.258	0.260	0.613	0.365	2.441
2	0.2	Tangential	3.942	0.174	0.393	0.327	2.613
		Cohesive	3.942	0.175	0.394	0.327	2.601
	0.3	Tangential	4.143	0.222	0.515	0.358	2.505
		Cohesive	4.142	0.222	0.515	0.358	2.503
	0.4	Tangential	4.255	0.259	0.611	0.366	2.445
		Cohesive	4.254	0.259	0.612	0.365	2.443
3	0.2	Tangential	3.467	0.115	0.220	0.224	2.897
		Cohesive	2.083	0.089	0.195	0.325	2.622
	0.3	Tangential	3.725	0.175	0.290	0.250	2.701
		Cohesive	2.243	0.112	0.254	0.354	2.514
	0.4	Tangential	3.887	0.177	0.349	0.347	2.696
		Cohesive	2.357	0.130	0.302	0.362	2.453
